# Reaching transgender populations in Zambia for HIV prevention and linkage to treatment using community‐based service delivery

**DOI:** 10.1002/jia2.25995

**Published:** 2022-10-12

**Authors:** Linah Mwango, Mona‐Gekanju Toeque, Brianna Lindsay, Kalima Tembo, Henry Sakala, Sean Reggee, Sibusiso Mimi Malunga, McLean Kabwe, Ina Kafunda, Adebayo Olufunso, Annie Mwila, Jackson Okuku, Nzali Kancheya, Kennedy Nkwemu, Daliso Mumba, Lottie Hachaambwa, Robb Sheneberger, Natalia Blanco, Marie‐Claude Lavoie, Kristen A. Stafford, Cassidy W. Claassen

**Affiliations:** ^1^ Ciheb Zambia Lusaka Zambia; ^2^ Maryland Global Initiatives Corporation Zambia Lusaka Zambia; ^3^ Center for International Health Education and Biosecurity University of Maryland School of Medicine Baltimore Maryland USA; ^4^ TransBantu Association Zambia Lusaka Zambia; ^5^ The Lotus Identity Lusaka Zambia; ^6^ Division of Global HIV and Tuberculosis U.S. Centers for Disease Control and Prevention Lusaka Zambia; ^7^ National AIDS/TB/STI Council Lusaka Zambia

**Keywords:** transgender people, HIV care continuum, Africa, HIV prevention, community, differentiated care

## Abstract

**Introduction:**

Transgender and gender‐diverse communities in Zambia are highly vulnerable and experience healthcare differently than cisgender persons. The University of Maryland, Baltimore (UMB) supports projects in Zambia to improve HIV case‐finding, linkage and antiretroviral treatment (ART) for Zambia's transgender community. We describe programme strategies and outcomes for HIV prevention, testing and ART linkage among transgender communities.

**Methods:**

UMB utilizes a differentiated service delivery model whereby community health workers (CHWs) recruited from key populations (KPs) reach community members through a peer‐to‐peer approach, with the support of local transgender civil society organizations (CSOs) and community gatekeepers. Peer CHWs are trained and certified as HIV testers and psychosocial counsellors to offer counselling with HIV testing and prevention services in identified safe spaces. HIV‐negative people at risk of HIV infection are offered pre‐exposure prophylaxis (PrEP), while those who test positive for HIV are linked to ART services. CHWs collect data using the standardized facility and community tools and a dedicated DHIS2 database system. We conducted a descriptive analysis examining HIV testing and prevention outcomes using proportions and comparisons by time period and geographic strata.

**Results:**

From October 2020 to June 2021, across Eastern, Lusaka, Western and Southern Provinces, 1860 transgender persons were reached with HIV prevention messages and services. Of these, 424 (23%) were tested for HIV and 78 (18%) tested positive. Of the 346 HIV‐negative persons, 268 (78%) eligible transgender individuals were initiated on PrEP. ART linkage was 97%, with 76 out of the 78 transgender individuals living with HIV initiating treatment. Programme strategies that supported testing and linkage included peer CHWs, social network strategy testing, same‐day ART initiation and local KP CSO support. Challenges included non‐transgender‐friendly environments, stigma and discrimination, the high transiency of the transgender community and the non‐availability of transgender‐specific health services, such as hormonal therapy.

**Conclusions:**

Peer KP CHWs were able to reach many members of the transgender community, providing safe HIV testing, PrEP services and linkage to care. Focusing on community gatekeepers and CSOs to disburse health messages and employ welcoming strategies supported high linkage to both PrEP and ART for transgender people in Zambia.

## 1 INTRODUCTION

Transgender denotes a diverse population of people whose birth sex does not correspond to their gender identity [1]. Globally, an estimated 25 million people identify as transgender and have some of the highest risks for HIV acquisition [1]. UNAIDS data estimate HIV risk is 13 times higher among transgender people, while studies have reported estimates of up to 50 times higher compared to the general adult population. Transgender people also have lower access to HIV services compared to other target populations [2, 3, 4]. Most research to date has focused on transgender women due to their disproportionate rates of HIV infection; transgender women who are sex workers are more likely to have HIV than cisgender female or male sex workers, with an estimated worldwide HIV prevalence of 27.3% [5]. A meta‐analysis found that laboratory‐confirmed HIV infection was significantly higher in transgender women (14.1%) compared to transgender men (3.2%) [6].

Factors associated with high HIV prevalence result from complex determinants of transmission risk, such as structural (e.g. violence, discrimination, stigma and social exclusion) and individual factors (e.g. drug dependence and anal sex without condoms) [1, 7, 8]. These interlocking factors impede progress at each stage of the HIV care continuum and prevent transgender populations from accessing services. HIV disease burden among transgender women and men is likely underestimated due to HIV programmes’ infrequent reporting on transgender persons, or from limited self‐identification in healthcare settings [1, 9]. These barriers impact opportunities for positive health outcomes, which could be improved through provisioning community‐friendly HIV treatment and prevention services [10]. HIV outcomes among transgender people are consistently poorer compared to cisgender populations, including lower rates of antiretroviral treatment (ART) adherence, retention in care, and viral suppression [11, 12, 13, 14].

Information on HIV's impact on transgender persons in sub‐Saharan Africa (SSA) is scant, as are acceptable, feasible and effective strategies to reach this population. In Zambia, HIV prevalence is estimated to be 11.1% among adults 15–49 [15]. Data on transgender populations are largely unavailable; however, UNAIDS estimates approximately 4000 transgender persons live in Zambia, of whom an estimated 38.6% have not yet been reached with HIV testing services (HTS) [16]. Despite transgender persons being disproportionally impacted by HIV, evidence‐informed strategies to optimize HIV care continuity remain scarce [17, 18]. In addition, transgender populations frequently relocate due to stigma and risks of criminalization, as Zambian law prohibits rights to exercise their sexuality and express their gender identity. Zambian laws criminalize same‐sex sexual acts for both men and women, which may be used to persecute transgender people. While sex work is not criminalized and Zambian law does not explicitly mention transgender persons, transgender people are often arrested on charges of loitering or vagrancy when practicing sex work [19, 20, 21].

The Community Impact to Reach Key and Underserved Individuals for Treatment and Support (CIRKUITS) and Zambia Community HIV Epidemic Control for Key Populations (Z‐CHECK) projects, implemented by the Center for International Health, Education, and Biosecurity (Ciheb) at the University of Maryland, Baltimore (UMB), and in collaboration with the Zambia Ministry of Health (MOH) and respective provincial health authorities, aimed to improve HIV case‐finding and linkage to care and treatment for key populations (KPs), including transgender people, at the community level in Zambia. The objectives of this paper are to present the CIRKUITS and Z‐CHECK approaches among transgender people in Zambia to HIV testing, treatment, and prevention services and to examine HIV positivity yield and ART linkage, with sub‐analyses by time period and province.

## 2 METHODS

### 2.1 Study design and setting

We conducted a retrospective analysis of aggregate programmatic data collected as part of routine CIRKUITS and Z‐CHECK service delivery from 1 October 2020 to 30 June 2021. Data included four of Zambia's 10 provinces—Lusaka, Southern, Western and Eastern—with estimated HIV prevalence of 15.1%, 12.4%, 10.6% and 7.4%, respectively [22]. Clients from all communities within catchment areas of 129 participating facilities were included.

### 2.2 CIRKUITS and Z‐CHECK interventions

CIRKUITS and Z‐CHECK are community HIV prevention and treatment programmes targeting key and priority populations. HIV services are delivered to residents of facility catchment areas by community health workers (CHWs) supervised by community liaison officers (CLOs) working with MOH healthcare workers (HCWs) in corresponding health facilities. CHWs must have completed secondary education; they are trained in HTS and psychosocial counselling. Following training, CHWs are assessed and certified as competent counsellors and HIV testers using MOH mentorship tools which are administered quarterly; they are recertified for HIV testing every 2 years. CLOs and CHWs are supervised by the public health facility staff in their catchment areas and report all community activities to the facility. Index testing, mobile testing, voluntary community testing and other HTS modalities, such as ad hoc and social network strategy (SNS) testing, were employed [23].

CHWs provided combination HIV preventive services, including social behavioural communication change, referral for voluntary medical male circumcision (VMMC), HIV pre‐exposure prophylaxis (PrEP), family planning (FP), and condom and lubricants distribution. Using a bidirectional referral system, HCWs, CLOs and CHWs direct clients to services based on medical conditions and preferences. Ninety‐four mobilizers, 287 CHWs and 55 CLOs were trained, mentored and deployed to conduct community mobilization and community‐based testing modalities, including index testing services and SNS (Table 1). Staffing included one technical lead, five CLOs and 53 CHWs who identified as KPs, including female sex workers (FSWs), men who have sex with men (MSM) and transgender persons.

**Table 1:** CIRKUITS and Z‐CHECK staffing across supported provinces and districts

Province	Estimated provincial HIV prevalence^a^	Provincial nurses	District	District CLOs	CIRKUITS/Z‐CHECK supported facilities	CHWs in district
Eastern	7.4%	1	Chipata	3	18	20
			Lundazi	3	8	10
			Petauke	3	7	10
Western	10.6%	1	Mongu	2	7	18
			Senanga	2	4	6
			Kaoma	2	3	6
			Kalabo	2	4	6
			Limulunga	0	1	2
Lusaka	15.1%	0	Lusaka Urban	16	17	45
			Chilanga	1	2	0
Southern	12.4%	1	Livingstone	4	13	41
			Kalomo	4	12	26
			Choma	4	12	31
			Monze	4	9	31
			Mazabuka	5	12	45
Total	Country: 11.1%	3		55	129	297

Abbreviations: CHW, community health worker; CLO, community liaison officer.

^a^ Prevalence data from ZDHS 2018 [22].

### 2.3 Approach to HIV case finding

**Figure 1:**
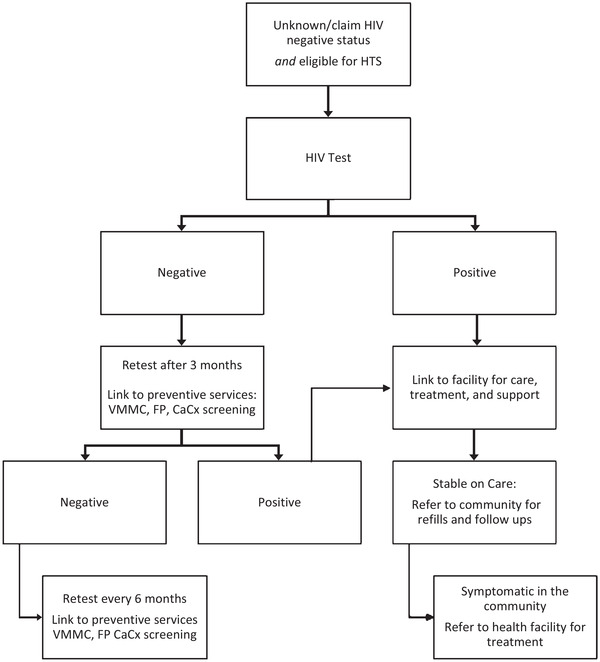
Community HIV testing services and ART linkage flow chart. Abbreviations: CaCx, cervical cancer; FP, family planning; HTS, HIV testing services; VMMC, voluntary male medical circumcision.

Working with local KP‐led civil society organizations (CSOs), including TransBantu Association Zambia and The Lotus Identity, we focused on communities where most transgender people are found and tailored our approach to include all gender‐diverse people, that is transgender men, transgender women, and non‐binary and gender‐non‐conforming individuals. Health facility staff, CLOs and CHWs used community hotspot mapping to identify locations where KPs gather, such as social events, guesthouses, nightclubs, brothels and lodges. The identified KP members could then access health services at community safe spaces. We established three community safe spaces in Lusaka and five in Southern Province. We trained HCWs in KP sensitivity, safety and security, PrEP and ART; HCWs then provided comprehensive healthcare services at safe spaces. Additionally, we trained transgender community members as community mobilizers; KP CHWs and mobilizers established networks with individuals belonging to KPs by sub‐type. Following prevention messaging services, CHWs offered HTS; persons interested in testing were screened with the MOH HIV risk screening tool and those with substantial risk (i.e. engaging in risk behaviours and have not tested for HIV within the past 3 months) were tested. Individuals who needed treatment or prevention services were linked to appropriate services at safe spaces or referred to health facilities for further management (Figure 1).

We employed safe and ethical index testing to identify people who did not know their HIV status [23]. Individuals who tested HIV positive were offered index testing services; all who accepted were screened for intimate partner violence (IPV) with the World Health Organization (WHO) IPV screening questionnaire prior to contact tracing. If individuals indicated a risk for IPV, this was documented and contact tracing was deferred. Persons at‐risk for IPV were referred to the “Gender‐Based Violence One‐Stop Center,” a structured system in Zambia where clinical, paralegal and psychosocial counsellors provide comprehensive care to IPV clients. For clients with a low risk of IPV who consented to partner notification services, the health provider and client agreed on a referral process [24]. Three WHO‐recommended approaches for index client partner notification were offered: (1) client referral: index client chooses to disclose their HIV status to sexual partners and suggest HTS; (2) provider referral: CHWs obtained consent from index client to contact sexual partners or biological children to offer HTS; and (3) dual referral: CHWs accompanied index client to assist with disclosure of HIV status and offer HTS to partner(s) and/or biological children.

### 2.4 Targeted community‐based testing modalities and linkage strategies

**Figure 2:**
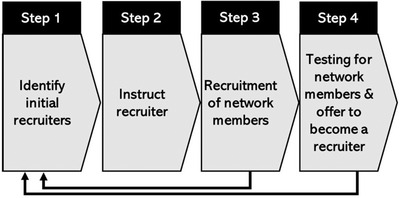
Diagram showing social network strategy recruitment strategy.

We provided community‐based HTS approaches, including SNS which provides HIV testing and counselling by using social network connections to locate individuals at the highest risk for HIV (Figure 2). SNS relies on the underlying assumption that people in the same social network share similar HIV‐risk behaviours [25]. Transgender people at risk of HIV acquisition received coupons to invite their networks to safe spaces or health facilities for the prevention or HTS. HIV‐negative persons were offered combination HIV prevention services consisting of condoms, FP, VMMC and PrEP. People who screened at substantial risk of HIV and tested HIV negative were offered and initiated on PrEP if eligible. HIV‐positive persons were offered same‐day ART by a clinical team of HIV nurse prescribers, pharmacists and psychosocial counsellors in the community or safe space. If facility HCWs were unavailable to join the clinical team, clients were escorted to the facilities by a CLO or CHW for ART initiation, and provided peer health navigation and adherence support to encourage sustained ART.

To optimize uptake and coverage of HIV testing, we provided tailored HIV/AIDS messaging for transgender men and women on HIV risks and benefits of early HTS. Working closely with the National AIDS/TB/STI Council, educational messages were developed and distributed in safe spaces; in addition, CLOs and CHWs offered pre‐ and post‐counselling for the transgender community. Using the peer‐to‐peer model and working with the transgender‐focused CSOs and community‐based mobilizers, we offered HIV prevention and HTS in hot spots predominantly frequented by transgender community members. Owners and managers of hotpots were oriented on the benefits of HIV prevention and HTS. All HTS was conducted confidentially according to national guidelines [26].

### 2.5 HIV testing and prevention outcomes

Outcomes of interest included: (1) the number of transgender persons reached with individual and/or small group‐level HIV prevention interventions; (2) the number of transgender individuals who accepted HTS; (3) the proportion of individuals tested; (4) the number of transgender persons identified HIV positive; (5) positivity yield, defined as the number of individuals newly diagnosed as HIV positive divided by the total number of individuals who received HTS and test results; (6) the number of transgender clients linked to ART among newly and previously identified HIV‐positive persons; (7) the proportion of individuals linked to treatment, defined as the total number of individuals newly enrolled and dispensed ART divided by the number of individuals newly diagnosed as HIV positive; and (8) PrEP uptake, the proportion of transgender individuals who were newly enrolled on oral PrEP among those who previously tested negative for HIV infection. For outcomes, we used definitions and guidance from the PEPFAR Monitoring, Evaluation and Reporting Indicator Reference Sheet (MER) version 2.3 [27]. We present results disaggregated by province and time period.

### 2.6 Data sources and collection

Aggregate data were abstracted from routine Zambian MOH HTS, PrEP and ART linkage registers. At the community level, each CHW collected patient‐level information onto individual community HTS and referral forms. At the facility level, CLOs then merged these data and entered them into facility registers. Aggregate data were manually entered into a customized District Health Information Software 2 data platform with internal data quality checks and validation rules.

### 2.7 Statistical analysis

Proportions were calculated for outcomes of interest, and the HIV care cascade analysed using descriptive statistics. Chi‐square tests were used to compare overall positivity yield and ART linkage by time period and province strata. All analyses were performed using *R* version 4.0.3.

### 2.8 Ethical approval

Ethical approval for this analysis was approved under the Z‐CHECK and CIRKUITS protocols by the ERES Converge Zambian Institutional Review Board (IRB) (2020‐Mar‐015, 2021‐Jan‐010), the Zambian National Health Research Authority (NHRA29/4/2020, NHRA0008/15/03/2021) and the UMB IRB (HP‐00086064, HP‐00096480). This project was reviewed in accordance with Centers for Disease Control and Prevention (CDC) human research protection procedures and was determined to be non‐research by the CDC. Informed consent was waived by the IRBs as this study was a retrospective review of aggregate programme data.

## 3 RESULTS

From October 2020 to June 2021, across 13 districts in Eastern, Lusaka, Western and Southern Provinces, 1860 transgender persons were reached with HIV prevention messages and services. The majority of transgender persons reached were in Lusaka Province, 81% (1505/1860), then Southern Province, 16% (304/1860) and only 3% (51/1860) in Eastern and Western Provinces combined.

**Figure 3:**
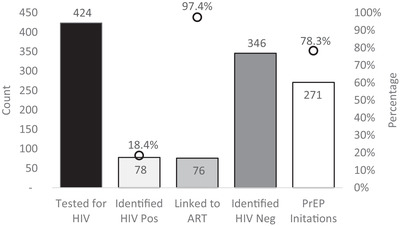
Transgender client prevention and treatment outcomes, Oct 2020–June 2021. Circles indicate HIV testing yield, linkage to treatment and PrEP uptake. Abbreviations: ART, antiretroviral therapy; HIV Neg, HIV negative; HIV Pos, HIV positive; PrEP, pre‐exposure prophylaxis.

Of transgender individuals who reached with HIV prevention messages, 424 (22.8%) were tested for HIV and 78 (18.4%) tested positive. Testing proportions ranged from 18.9% to 24.7% by quarter and were the highest in Eastern Province (68.8%). ART linkage was high overall with 76 (97.4%) initiating treatment (Figure 3). Positivity yield did not vary significantly by province (*p* = 0.21); however, there were low numbers tested in Eastern and Western Provinces (Table 2). In Lusaka Province, yield was 17.4% (95% CI 13.6–22.0%) compared to 16.0% (95% CI 9.6–25.5%) in Southern Province. Of the 346 HIV‐negative transgender persons, 268 (77.5%) initiated PrEP. PrEP uptake did not vary significantly by province, but was 80.1% (95% CI 74.8–84.5%) in Lusaka (209/261) and 61.8% (95% CI 49.9–72.4%) in Southern (42/68) (Table 2).

**Table 2:** Prevention and treatment outcomes by province and quarter, Oct 2020–June 2021

	Reached	Tested	Testing rate	HIV positive	Yield	Linked	Linkage	HIV negative	PrEP	PrEP
	*n*	*n*	(%)	*n*	(%)		(%)	*n*	*n*	(%)
**Province**										
Eastern	32	22	68.8	10	45.5	10	100.0	12	12	100.0
Lusaka	1505	316	21.0	55	17.4	55	100.0	261	209	80.1
Southern	304	81	26.6	13	16.0	11	84.6	68	42	61.8
Western	19	5	26.3	0		0		5	5	100.0
**Quarter**										
Oct to Dec 2020	866	214	24.7	46	21.5	44	95.7	168	122	72.6
Jan to Mar 2021	557	105	18.9	16	15.2	16	100.0	89	78	87.6
Apr to Jun 2021	437	105	24.0	16	15.2	16	100.0	89	68	76.4
Overall	1860	424	22.8	78	18.4	76	97.4	346	268	77.5

Abbreviation: PrEP, pre‐exposure prophylaxis.

October to December 2020 demonstrated the highest number of transgender individuals reached with HIV prevention messages and the highest percentage of HIV testing yield (21.5%). ART linkage was consistently high over all three quarters (Table 2). The absolute number of PrEP initiations was highest from October to December 2020; however, PrEP uptake was highest between January and March 2021.

## 4 DISCUSSION

We report programmatic achievements across the CIRKUITS and Z‐CHECK projects in Zambia, which used community mobilizers and peer navigators, including transgender CHWs and CLOs, to reach transgender individuals with HIV services. Although uptake of HTS was modest (22.8%), overall HIV positivity yield was 18%, with 97% linkage to ART. Linkage to PrEP among transgender individuals who tested negative for HIV was 78%.

HIV positivity yield among transgender clients in this study was above the national adult prevalence of 11–12%. However, this yield was relatively low compared to other effective general population testing strategies, such as index testing (45%) [23] and HIV prevalence among other KP in Zambia, for example 21.1% among MSM, 42.2% among FSW and 28.0% among incarcerated persons [23, 28]. Regional data on transgender HIV positivity rates are scarce; however, our finding of 18.4% HIV positivity among all transgender people was consistent with the global HIV prevalence of 19% among transgender women [4] but lower than the estimated prevalence among transgender women in SSA, which may range from 25% to 43%, with an incidence rate from 4.7 to 20.6 infections per person‐years [4, 29].

Our HIV yield data are limited by low uptake of HTS, meaning that most transgender persons declined HIV testing. Low testing may have been due to ineligibility: per MOH guidelines, people being tested for HIV must be deemed at substantial risk using the MOH HIV risk screening tool. Thus, not everyone who is offered HTS is tested. It remains unclear whether HIV prevalence among transgender‐identifying individuals is lower in Zambia, or if we are missing a critical portion of the HIV‐positive transgender population, especially if those declining to be tested are likely to be HIV positive, or if some KP clients were not correctly classified as transgender.

We employed several key strategies to reach transgender persons, including partnering with local transgender CSOs, TransBantu Association Zambia and The Lotus Identity. These CSOs provided introductions to local KP networks and helped access members of the transgender community. They also trained our staff and HCW in KP sensitivity, safety and security, especially around the unique concerns and health needs of transgender persons. Second, we employed peer navigators to help reach the transgender community. Local KP CSO involvement was critical to success as it ensured a trusted member of the community was bringing services. Other studies using similar peer navigation networks to reach the community have also demonstrated success [30, 31].

Data showed an initial push of increased volume during the first quarter of implementation with lower volume but sustained testing yield and linkage, perhaps owing to an outlet for unmet need. Of note, this time period also overlaps with the second significant wave of COVID‐19 in Zambia, potentially impacting service delivery.

We used targeted HTS strategies and while we were unable to disaggregate data by modality, our programmatic implementation focused on SNS and index testing. SNS has been an effective strategy in reaching the transgender community, as social network members know best where to find other community members. Other studies using SNS testing in SSA have proven cost‐effective and increased the identification of hard‐to‐reach undiagnosed HIV‐positive KPs [32, 33, 34].

ART linkage was moderate; however, it compares favourably to other rates of linkage among KPs in the region. For example, a study from Tanzania found linkage rates of 78% [35], and other studies reported rates as low as 55% [36, 37, 38, 39, 40]. We attribute this programmatic achievement to several key strategies. First, we provided high‐quality training in KP and transgender‐specific health needs and sensitivity to key healthcare personnel, who then worked with our programmes and transgender community members. These HCWs provided care in a non‐stigmatizing or non‐discriminatory manner and came to be trusted by the transgender community. Second, we provided same‐day initiation of ART to people who tested positive in the community, which helped to ensure high linkage. Finally, we provided ART refills at healthcare centres, community safe spaces and via home delivery by the CHWs. HCWs would visit offsite locations and provide refills, which also served as a reminder for medication adherence.

Our programme demonstrated remarkable achievements in PrEP, with 78% uptake of PrEP among transgender clients. This constitutes one of the first estimates of PrEP linkage for transgender persons in SSA. Other studies in the region have shown PrEP uptake of 81% in Kenya; lower rates have been reported in South Africa with uptake at 16% which underscores the existing challenges [41, 42]. Livingstone and Lusaka were the first areas to begin PrEP implementation, enabling a robust healthcare infrastructure to support PrEP to specifically engage the transgender community. Transgender peer navigators provided counselling about the benefits of PrEP and shared their personal experiences on PrEP. Being able to obtain refills via the community safe spaces likely improved PrEP uptake and persistence [43].

We faced several challenges to programme implementation. Although reasons for not testing were not routinely collected, counsellors reported anecdotally that KP members, including gender‐diverse individuals, are exhausted from being tested for HIV. They are tested often and feel like the international donor community is not addressing their other health concerns, particularly around the unavailability of transgender‐affirmative health services, such as hormonal therapy or counselling. This may have led to the relatively low uptake of HTS among those who were provided HIV prevention messages. Future efforts should include transgender‐related healthcare, such as hormonal therapy and mental health services.

Second, the transgender population is highly mobile as they continue to face criminalization, which creates challenges to viral load coverage, ART adherence and PrEP persistence. Highly unfriendly environments exist for transgender people, leading to stigma and discrimination.

Finally, most of our success in reaching transgender men and women was limited to the urban areas of Lusaka and Livingstone. This geographic concentration may be for several reasons: (1) members of the transgender community willing to identify as transgender may be more likely to reside in and identify as transgender at a greater frequency in urban areas compared to rural areas; (2) limited transgender friendly service opportunities exist in rural versus urban areas; (3) rural parts of Zambia may be more influenced by local tradition, norms and culture, with less awareness of transgender identities; and/or (4) our transgender peer navigators were based in Lusaka, and thus we had the greatest success in Lusaka compared to non‐transgender peer navigators located outside of Lusaka.

The primary limitation of this study is that clients had to identify as transgender for inclusion in our analysis. Due to stigma, we may have underestimated the number of transgender‐identifying individuals reached with prevention and treatment services. We were unable to disaggregate the study population by identification as transgender men, transgender women, non‐binary or gender‐non‐conforming, which would help to shed more light on populations being reached under these projects. Similarly, our HIV testing proportion includes all individuals who were reached with prevention messages in the denominator; data were not available on reasons for declining HTS. Therefore, the tested proportion is best interpreted with caution as we are not able to ascertain if individuals were not tested because they had been tested within the previous 3 months or declined for other reasons. These results also do not include HIV self‐testing, so reach with HTS is likely higher. We also note the lack of both ART and PrEP persistence data, so we cannot gauge how many people remained on PrEP and ART after initial uptake.

Finally, episodic COVID‐19 waves during programme implementation potentially affected both project achievement and client uptake of services. During periods of increased COVID cases, there were restrictions on the number of people at health facilities, causing some clients to miss lab, pharmacy or clinical appointments. Furthermore, global logistics challenges resulted in shortages of PrEP drugs. The programme was adapted by having CHWs deliver services and commodities to clients in the community as much as possible.

## 5 CONCLUSIONS

The UMB peer navigation model was able to reach many transgender community members with HIV services in Zambia. Engaging local transgender CSOs is critical to establishing trust and reaching community members. Index testing and SNS are effective high‐yield testing strategies for reaching transgender persons. Using sensitized HCWs and community safe spaces enhanced ART linkage and PrEP uptake among the transgender population. Moving forward, the inclusion of transgender‐specific health services can be considered a best practice for programmes supporting the transgender community.

## COMPETING INTERESTS

The authors declare that they have no competing interests.

## AUTHORS’ CONTRIBUTIONS

All authors contributed to writing, reviewing and editing the manuscript. All authors have read and approved the final manuscript.

## FUNDING

The CIRKUITS and Z‐CHECK projects and this publication are supported by the U.S. President's Emergency Plan for AIDS Relief (PEPFAR) through the U.S. Centers for Disease Control and Prevention (CDC), Division of Global HIV and Tuberculosis (DGHT), and in collaboration with the Ministry of Health of Zambia, under the terms of NU2GGH002123‐01‐02 and U2GGH001913. CDC investigators did not interact with human participants or have access to identifiable data or specimens for research purposes.

## DISCLAIMER

The views expressed in the manuscript do not necessarily represent the views of CDC. The findings and conclusions in this manuscript are those of the authors and do not necessarily represent the official position of the funding agencies.

## Data Availability

The data that support the findings of this study are available upon reasonable request from the corresponding author. The data are not publicly available due to privacy or ethical restrictions.
